# The factors affecting a native obligate parasite, *Cuscuta australis*, in selecting an exotic weed, *Humulus scandens*, as its host

**DOI:** 10.1038/s41598-018-36997-7

**Published:** 2019-01-24

**Authors:** Ai-Ping Wu, Wen Zhong, Jin-Rui Yuan, Liang-Yu Qi, Fa-Lin Chen, Yun-Shan Liang, Fei-Fei He, Yan-Hong Wang

**Affiliations:** 1 Ecology Department, College of Bioscience and Biotechnology, Hunan Provincial Key Laboratory of Rural Ecosystem Health in Dongting Lake Area, Hunan Agricultural University, Changsha, 410128 China; 2 School of Agriculture, Yunnan University, Kunming, 650091 China; 3 School of Forestry and Bio-technology, Zhejiang Agriculture and Forestry University, Hangzhou, 311300 China; 4 Department of Biology, Lund University, SE-223 62 Lund, Sweden

## Abstract

In weed management, using native parasites to control exotic weeds is considered a better alternative than classical biological control. But the risk must be assessed because of the potential damage caused by these agents. We conducted this project to investigate the mechanism driving the choice of a native obligate parasite, *Cuscuta australis*, between the exotic, *Humulus scandens*, and native plants as its host through field and pot experiments. The results showed that *C. australis* preferred the exotic weed over native (naturalized) hosts and caused a notable reduction in the biomass of *H. scandens* in the field. In contrast, the results of the pot experimentindicated that *C. australis* preferred a mix of native (naturalized) hosts over the exotic weed. Both texperiments indicated that the parasitic preference of *C. australis* was induced more by light irradiance than plant water, carbon (C), nitrogen (N) and phosphorus (P) contents, indicating that the native parasite can only be used to control *H. scandens* when the exotic weed forms mono-cultures or dominates the community. Accordingly, induction and release of *C. australis* to control *H. scandens* should be conducted with great caution.

## Introduction

Classical biological control has long been considered a “green” alternative in weed management due to its advantages in terms of effectiveness, cost, persistence and environmental friendliness^1,2^. However, the use of introduced biocontrol agents in the management of invaded ecosystems remains controversial because of attacks on nontarget species, negative effects on ecosystems and secondary invasion^3,4^ and screening an appropriate agent in its home range prior to its introduction and release takes a long time^5^. Therefore, native natural enemies are currently recommended as potential agents for the biocontrol of exotic weeds^5^. Native enemies are superior to introduced species because they have coevolved with native species and have adapted to the local plant phenology, which minimizes the negative impacts on nontarget species and the whole ecosystems^6^. Some particular native natural enemies, parasites, have been viable and effective biocontrol agents for some exotic invasive plants in many ecosystems^7–9^, and studies have demonstrated that native parasites prefer invasive hosts over native hosts and cause great damage to exotics. However, which factors cause parasites to prefer exotic weeds over native hosts remains poorly understood.

Generally, light irradiance and nutrient concentration have been considered the two main factors affecting the host choice by a parasite (such as dodders, genus *Cuscuta*). It is generally thought that the location of a host and subsequent attachment by a dodder are mainly induced by changes in light quantity and quality rather than by volatile chemical cues from host plants^9,10^. *Cuscuta* seedlings conspicuously grow toward conditions with low red light:far-red light (R:FR) ratios, which are associated with denser canopy environments^9,10^, so the probability of encountering and parasitizing a host plant is much greater for dodder seedlings, which is consistent with previos results that the spread of parasites is mainly driven by host density^11,12^. Furthermore, parasites often prefer hosts with higher nutrient contents, especially nitrogen (N) (such as legumes) as N content is important in parasite performance, although it is not always better on nutrient-rich plants^13–16^. In addition, generalist parasitic plants deliberately parasitize a mixture of host species to either obtain various types and amounts of nutrients or minimize the toxic effects of a single host^17^. Accordingly, it is very possible that parasitic plants would select a combination of plants with higher nutrient contents as their preferred hosts.

In native communities, parasitic plants can increase species biodiversity by parasitizing and suppressing competitively superior and dominant host species^18^. Exotic invasive plants are usually the most competitive and dominant species in invaded communities^19^. So parasitic plants may prefer to parasitize and reduce the coverage of these first-order competitors (exotic plants in invaded communities) as keystone species and ecosystem engineers^20,21^. Experimental and observational results have actually showed that some exotic plants were more susceptible to these novel native generalist parasites than native and naturalized plants in their invaded ecosystems^7–9^, possibly because these invasive species have not evolved to resist or mitigate the virulence of the parasites^21,22^. However, whether the parasitic preference for exotic invasive plants is caused by the lower red light:far-red light ratio under the dense canopy (and/or higher nutrient contents) of invasive plants or the origin of the host requires more extensive research.

**Figure 1:**
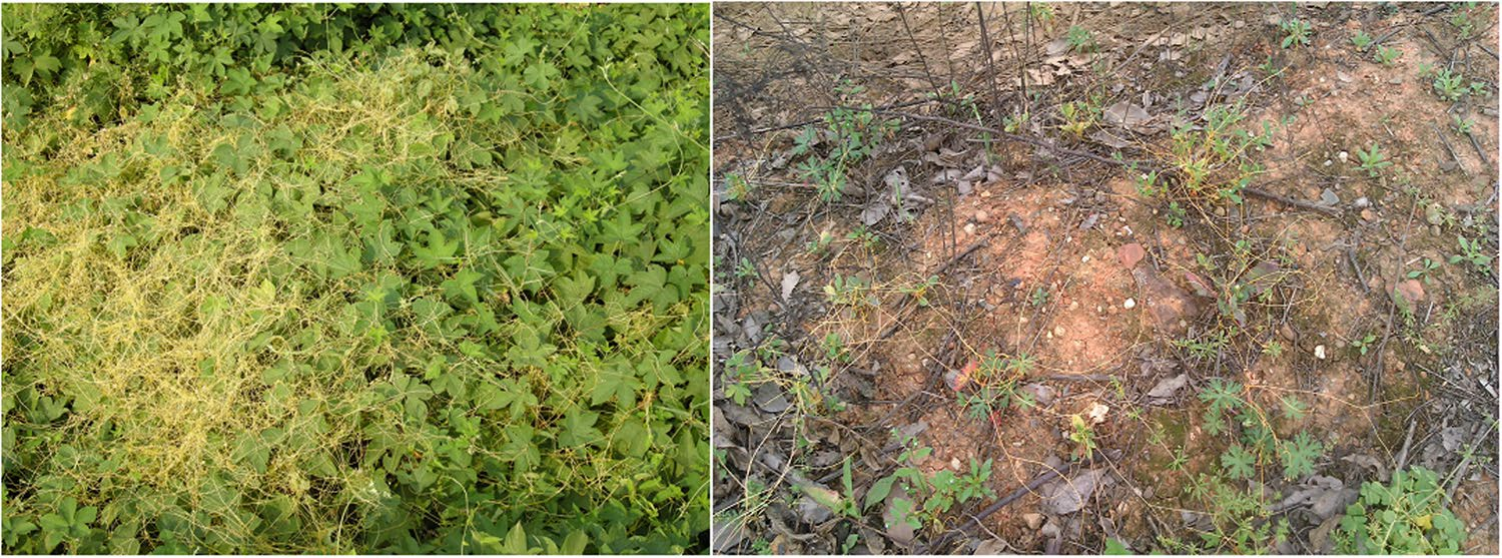
Native obligate parasite *C. australis* parasitizes exotic weed *H. scandens* (left) and other native plants (right) in the field. Photo by Aiping Wu.

We previously found that a native obligate parasite, *Cuscuta australis* R. Br., was able to parasitize and suppress an exotic weed, *Humulus scandens*, in a field survey (Fig. 1), so our goal in this study was to compare the preferences of parasite for native and exotic plants and assess the risk of using this potential biocontrol agent before its induction and release. We hypothesized that 1) *C. australis* would prefer exotic *H. scandens* over single native (naturalized) hosts because *H. scandens* has higher nutrient contents and an exotic origin; 2) *C. australis* would prefer a mixture of native (naturalized) hosts over the exotic *H. scandens* due to greater benefits and 3) the host preference of dodders would mainly be induced by light cues resulting from the plant canopy rather than the properties of the host plant.

## Results

### Field experiment

**Figure 2:**
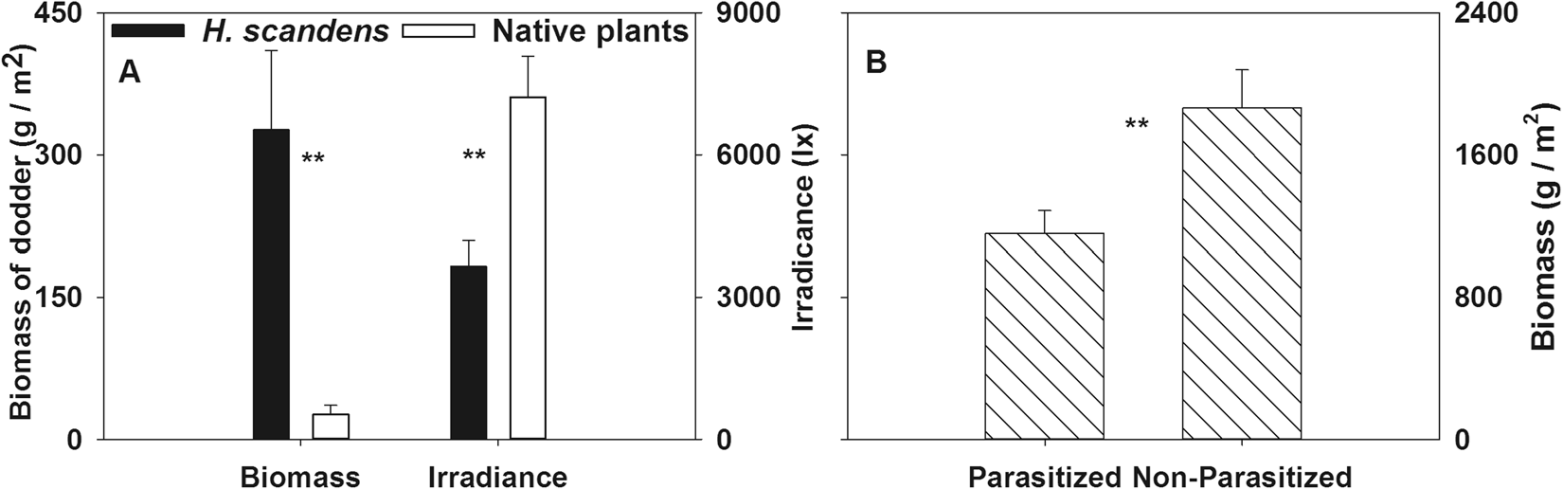
Dodder’s biomass and light irradiance in native and parasitized *H. scandens* sub-communities (**A**) and biomass of *H. scandens* in parasitized and non-parasitized sub-communities (**B**). **Means significant different at p < 0.01.

In the field survey, the dodder biomass in the parasitized *H. scandens* subcommunity was significantly higher (74.4%) than that in the native subcommunity, while the light irradiance under the canopy of the parasitized *H. scandens* subcommunity was much lower (49.4%) than that under the canopy of the native subcommunity (Fig. 2A, ANOVA, p < 0.01). The aboveground biomass of *H. scandens* in the parasitized subcommunity was much lower (37.8%) than that in the nonparasitized subcommunity (Fig. 2B, ANOVA, p < 0.01).

### Pot experiment

**Figure 3:**
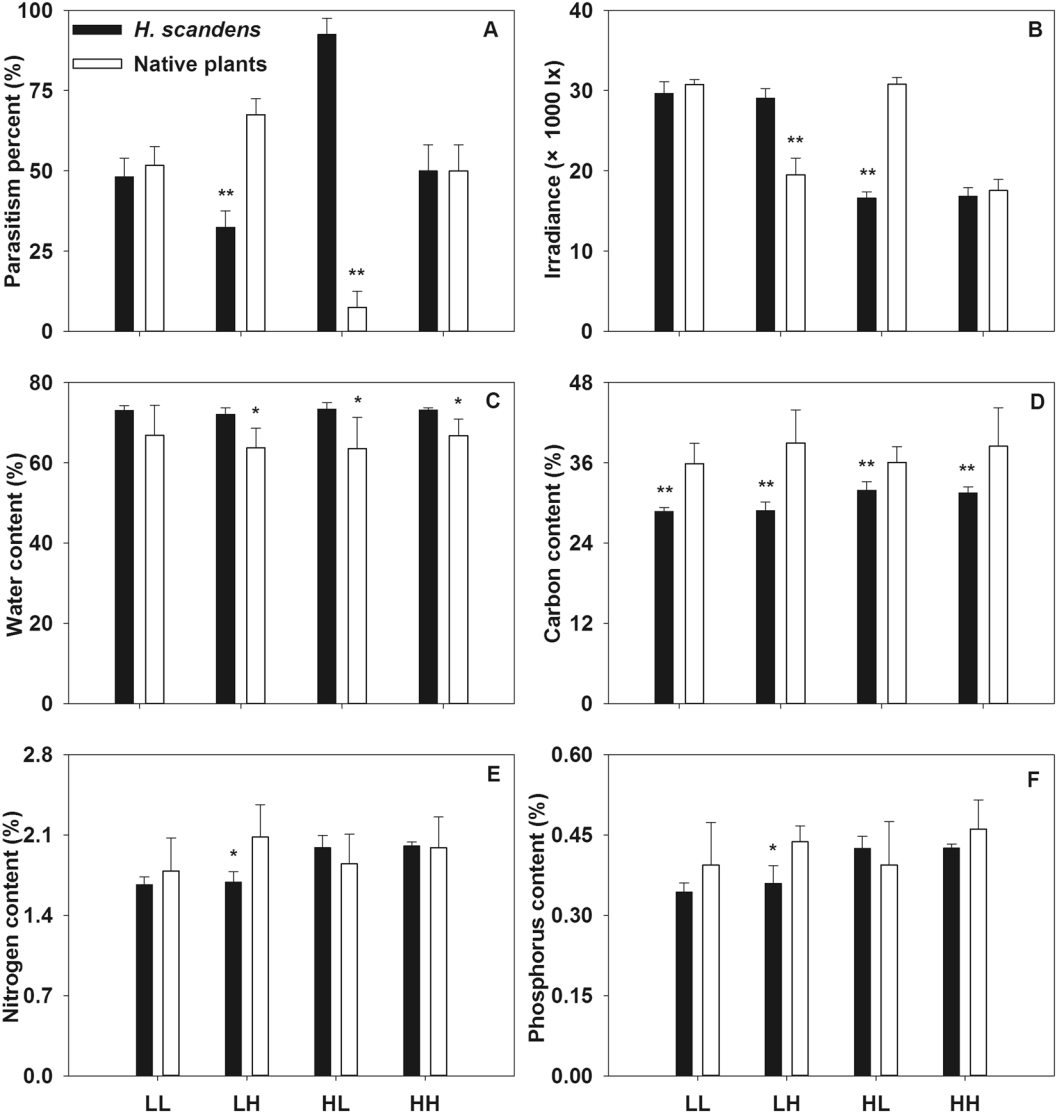
Parasitic percent (**A**), irradiance (**B**), water content (**C**), carbon content (**D**), nitrogen content (**E**) and phosphorus content (**F**) of native and exotic sub-communities in the four different density modes with single native host treatment. *Means significant different at p < 0.05, **Means significant different at p < 0.01.

**Figure 4:**
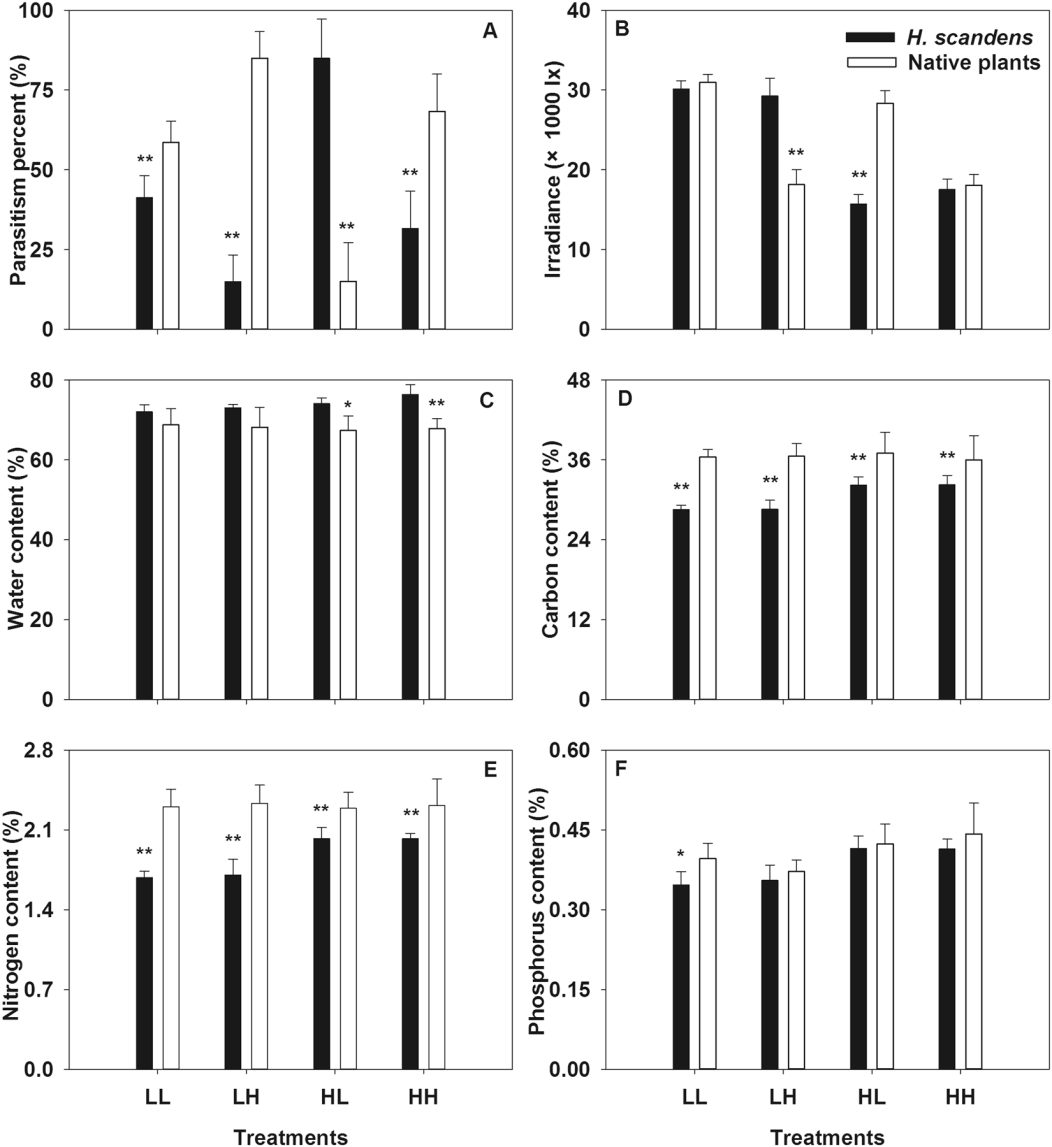
Parasitic percent (**A**), irradiance (**B**), water content (**C**), carbon content (**D**), nitrogen content (**E**) and phosphorus content (**F**) of native and exotic sub-communities in the four different density modes with mixed native host treatment. *Means significant different at p < 0.05, **Means significant different at p < 0.01.

The results from the single and mixed native (naturalized) host treatments were similar in the pot experiment (Figs 3 and 4). For the single native host treatment, the percent parasitism was not significantly different between the native and exotic subcommunities in the LL and HH treatments, but the percent parasitism in the *H. scandens* subcommunity was distinctly lower in the LH treatment and higher in the HL treatment relative to the native subcommunity (Fig. 3A, ANOVA, p < 0.01). Light irradiance did not significantly different between the native and exotic subcommunities in the LL and HH modes, but that in the *H. scandens* subcommunity was higher in the LH mode and lower in the HL mode compared to the native subcommunity (Fig. 3B, ANOVA, p < 0.01). The water content of *H. scandens* was higher than that of native plants in three of the planting density modes (no differences in the LL mode), whereas the C content exhibited an opposite pattern (Fig. 3C,D, ANOVA, p < 0.05). The N and P contents were both higher in native plants than in exotic plants in the LH mode, while they did not differ markedly between native and exotic plants in the other three modes (Fig. 3E,F, ANOVA, p < 0.05). The results of two-way ANOVA showed that the differences in percent parasitism were mainly caused by density rather than plant origin or the interaction of the two factors (Table 1, ANOVA, p < 0.01).

**Table 1:** ANOVA results of origin (native vs. exotic) and density (low vs. high) effects on the parasitic percent of dodder in different native host treatments.

Source	Single native host treatment			Mixed native host treatment		
	*df*	F	p	*df*	F	p
Origin (O)	1	3.163	0.086	1	4.522	**0.039**
Density (D)	1	21.129	**0.000**	1	30.543	**0.000**
O × D	1	0.019	0.892	1	0.591	0.446
Residual	28			44		

Values of P < 0.05 are in bold.

In the mixed native host treatment, percent parasitism in the exotic subcommunities was higher in the HL mode, whereas it showed the opposite pattern in the other three modes (Fig. 4A, ANOVA, p < 0.01). Light irradiance, water content (except in the LH mode) and C content were the same as those in the single native host treatment (Fig. 4B–D, ANOVA, p < 0.01). The N content was higher in native plants than in exotic plants in all modes, and the P content was higher in native plants than in exotic plants in the LL mode but not evidently different in the other three modes (Fig. 4E,F, ANOVA, p < 0.05). The results of two-way ANOVA showed that the differences in percent parasitism were caused by density and plant origin but not an interaction between the two (Table 1, ANOVA, p < 0.05).

### Stepwise multiple regression analysis

The Pearson correlations among the variables showed that the percent parasitism by dodder was not significantly related to the plant water (Table 2, R = 0.014, p = 0.901), or C (Table 2, R = 0.193, p = 0.086) and P (Table 2, R = 0.195, p = 0.084) contents, but the relationships between the percent parasitism by dodder and the plant N content (Table 2, R = 0.314, p = 0.005) and irradiance (Table 2, R = −0.582, p = 0.000) were both significant. However, the results of stepwise multiple regression analysis showed that only irradiance could be best fitted to the percent parasitism data, and the adjusted R^2^ value was 0.330 (Table 3).

**Table 2:** Pearson correlation matrix of different variables.

	Percent parasitism	Irradiance	Water content	C content	N content	P content
Percent parasitism	1	−0.582**	0.014	0.193	0.314**	0.195
Irradiance		1	−0.162	−0.170	−0.347*	−0.476**
Water content			1	−0.429**	−0.168	−0.160
C content				1	0.529**	0.308**
N content					1	0.230*
P content						1

Statistically significant correlation coefficients are highlighted in bold, *p < 0.05, **p < 0.01.

**Table 3:** Stepwise multiple regression analysis for prediction of percent parasitism using the original independent variables.

Predictors	Constant	Irradiance
Adjusted R square		0.330
Estimated regression coefficient	107.185	−2.416
Standard Error	9.369	0.382
p	0.000	0.000

## Discussion

Firstly, we report that a native parasite, *C. australis*, can parasitize and suppress the exotic weed, *H. scandens*, and it can cause substantial biomass loss (37.8%) of the exotic weed in the field, which indicates that *C. australis* can be used as a potential agent for the biocontrol of *H. scandens*. However, the parasitic preferences between native plants and exotic weeds were distinctly opposite in the two experiments: *C. australis* was observed to prefer exotic weeds over native hosts in the field, while it preferred mixed native hosts over exotic hosts in the pot experiment. Therefore, identifying the causes of the differences in parasitic preferences addresses the knowledge gap of the potential use of *C. australis* to control *H. scandens*.

Because *C. australis* can parasitize both types of plants to a great extent, we can speculate that its parasitic preferences are not caused by the defenses of native and exotic plants or by its own detoxification mechanism. Generally, parasites are thought to prefer hosts with higher N contents^13–16^, and although our results also showed that the percent parasitism by *C. australis* is significantly related to higher plant N content (Table 2) that is not the main cause. In our pot experiment, *C. australis* preferred the exotic weed over native hosts in the HL mode, while the N contents of the exotic plants were lower than those of native plants in the mixed native host treatment (Fig. 4A,E). Furthermore, stepwise multiple regression analysis indicated that parasitic preference of *C. australis* was not primarily caused by plant N content (Table 3). Accordingly, plant N content is not the main determinant of parasitic preferences in *C. australis*. Similarly, the parasitic preferences are also not induced by plant water, or C or P contents because percent parasitism by *C. australis* was not significantly correlated to these indexes (Tables 2 and 3). Furthermore, this parasite prefers mixed native hosts, enen though *H. scandens* had a higher water content and lower carbon (C) content in the mixed native host treatment (Fig. 4A,C,D, Table 1), so it can be concluded that the preferences of *C. australis* are mainly caused by factors other than plant properties. Luijckx *et al*. also concluded that parasite infectivity largely depends on factors other than host nutrients at the beginning of the infection process^23^.

Our data indicated no parasitic preference between native and exotic hosts occurred in the single native host treatment, whereas there was a significant parasitic preference for mixed native hosts in the mixed native host treatment (Table 1). Parasitic preferences for mixed hosts indicate that the parasite can improve its nutrient balance and minimize toxic effects, which is consistent with previous findings^20^. In addition, a mixed diet increases the ability of a parasite to encounter high-quality hosts by supporting growth in the gaps between preferred plants^17^, and parasites can obtain more nutrients from mixed hosts because of the increasing nutrient availability and production provided by their mutualism^24^, which is consisted with the higher nutrient contents (especially N) in the mixed native hosts observed in our study (Figs 3 and 4). Even though we did not investigate this factor in this experiment, another advantage of mixed hosts is that parasites can protect themselves to some extent from suffering the effects of environmental stressors, particularly herbivory^25^.

The results show that the percent parasitism by *C. australis* is significantly negatively related to irradiance under the plant canopy and that the plant density greatly affects the percent parasitism (Tables 1–3), supporting the results from the field experiment (Fig. 2A), and these findings highlight that the parasitic preferences of *C. australis* are more affected by light irradiance than by plant properties^10,25^. High plant density and low irradiance indicate a dense plant canopy^9,10^, which is especially prominent among exotic plants in invaded communities^19^. Thus, the probability of *C. australis* encountering and parasitizing a host plant is much higher if it grows toward such environments^11,12^. In addition, the percent survival of *C. australis* will increase because the successful search for a suitable host plant is the most important step in its survival due to the limited resources available to its seedling^10^.

Parasites prefer hosts with a dense canopy (low irradiance) in the ecosystem regardless of whether the nutrient contents in the hosts are high or low, as observed in our field and pot experiments, which not only increases the survival rate of parasites but benefits them to a much greater extent. The total nutrient quantity (at least per unit area) in the dominant host is very likely greater than that in all other plants due to its high biomass, so it can support more parasites and sustain them for a longer time^13,26^. In addition, for parasites with low migration capacities, their spread can also be promoted by host density^11,12^. Moreover, competitive and dominant plants (especially exotic plants) usually have higher growth rates, and parasite performance is strongly correlated to the growth rate of the hosts^27^. As the most competitive and dominant plants in an ecosystem, exotic plants are more likely to be encountered by the seeds and seedlings of parasites^16^. Therefore, we can conclude that parasitizing dominant and competitive exotic hosts provides many advantages for a parasite, and the probability of a parasite encountering exotic weeds is much greater in invaded ecosystems. Both of these factors contribute to the parasitic preference of *C. australis* for the exotic weed *H. scandens* in our field study^13,20^.

In summary, *C. australis* prefers the exotic weed *H. scandens* over native hosts and causes great biomass reduction in the field, but the pot experiment indicates that *C. australis* prefers mixed native hosts over the exotic weed and that parasitism is caused more by light irradiance than by plant properties. Accordingly, we suggest that the induction and release of *C. australis* to control *H. scandens* should only be used when *H. scandens* forms mono-cultures or solely dominates a community.

## Materials and Methods

### Field experiment

In late autumn 2014, we selected six *H. scandens* communities parasitized by *C. australis* in the city of Changsha, Hunan Province. In each community, three subcommunities were identified located at least 10 m apart: a parasitized *H. scandens* subcommunity, a nonparasitized *H. scandens* subcommunity and a native subcommunity (including a naturalized species, *Medicago sativa*). To assess the suppressing effects of *C. australis* on the *H. scandens* community, three 1.0 × 1.0 m^2^ plots were established in each of the parasitized and nonparasitized *H. scandens* subcommunities to measure the aboveground biomass of *H. scandens* after removing (only for the parasitized subcommunity) all *C. australis* organs from the *H. scandens*. Similarly, to compare the parasitism preference between *H. scandens* and native (naturalized) species, three 1.0 × 1.0 m^2^ plots were also established in each of the parasitized and native subcommunities and dodder biomass on the plants and light irradiance under the plant canopy were measured in each plot. The dodder biomass was weighed after collecting all *C. australis* organs dissected from the stems and leaves of hosts. Light irradiance under the plant canopy in each plot was measured three times, and the mean value was considered to be the irradiance of the plot.

### Pot experiment

From April to June 2014, a pot experiment was conducted in a greenhouse at Hunan Agricultural University, Changsha, China. In April 2014, seedlings of the exotic weed *H. scandens* and four co-occurring native (naturalized) species were selected from an abandoned field (113°4′25.39″E, 28°11′3.92″N)that included *Artemisia rubripes*, *Geranium carolinianum*, *Medicago sativa* and *Polygonum perfoliatum*, all of which can be parasitized by *C. australis* in the field (Fig. 1). To compare parasitic preferences between native and exotic plants, an exotic subcommunity and a native subcommunity were constructed and planted separately (spaced 10 cm apart) on the both sides of a pot. Each pot had a rectangular area of 1500 cm^2^ (soil surface), a depth of 20 cm and 25 kg of paddy soil (organic matter: 20.2–26.7 g kg^−1^, total N: 6.2–6.67 g kg^−1^, total P: 0.52–0.67 g kg^−1^ and pH: 5.86–6.12, soil layer approximately 15 cm). Four density modes were established: low-density exotic subcommunity vs. low-density native subcommunity (LL), low-density exotic subcommunity vs. high-density native subcommunity (LH), high-density exotic subcommunity vs. low-density native subcommunity (HL) and high-density exotic subcommunity vs. high-density native subcommunity (HH). Four individual plants were planted in the low-density subcommunities, and eight individual plants were planted in the high-density subcommunities. At the same time, two types of native subcommunity were established: only one native (naturalized) species was planted, which was treated as the single native host treatment; and all four native (naturalized) species were planted together, which was treated as the mixed native host treatment. The plants were evenly planted on both sides of the pots, and each native host treatment was replicated six times for the mixed native host treatment and four times for the single native host treatment. We measured light irradiance under the plant canopy of each subcommunity in triplicate twenty days after the hosts were planted, and the mean value was considered the light irradiance under the subcommunity. Simultaneously, ten pregerminated *C. australis* seedlings (approximately 4 cm with ring-like bases) were carefully and evenly placed in the center of the space between the native and exotic subcommunities in each pot, and the tip of each seedling was gently manipulated to point upward. In this experiment, we used 4 density modes, 2 native host treatments, and six or four replicates, resulting in a total of 40 pots. To prevent plants (especially the vines) from growing toward one another, a bamboo cane (80 cm long) was placed vertically beside each plant to support it. During the experiment, 300–500 ml of tap water was added to each pot every morning (7:00) to maintain soil moisture. Weeds (all unintentionally cultivated plants) were carefully removed from the pots, and other appropriate care was taken to minimize any disturbance. The survival and parasitic success of *C. australis* were considered to have occurred only when the tip extended more than 5 mm outward from the stem or remained coiled. The percent parasitism by *C. australis* in each subcommunity was determined one month after the planting, and it was calculated as the total number of surviving *C. australis* in each subcommunity divided by the total number of surviving individuals in each pot. Then, the shoots of each species were harvested and weighed after removing all *C. australis* organs from the stems and leaves of the hosts in each pot. The shoots were then oven dried at 65 °C for 72 h after drying at 105 °C for 30 minutes, weighed, and processed into fine powder for analysis. The water content of the plants was measured by dividing the weight of the water (wet weight minus dry weight) by the wet weight.

### Laboratory analysis

The total N concentrations (% dry mass) of the shoots were determined using an automatic N analyzer (Büchi-339), and the total P (% dry mass) contents were analyzed using the vanadium molybdenum yellow colorimetric method after digestion in H_2_SO_4_ and H_2_O_2_ and standardization against known reference materials. The total C concentrations (% dry mass) of the shoots were measured using a TOC analyzer (WIN TOC1010, O. I. Corporation, USA) after combustion at 650 °C with glass fiber.

### Data analysis

All the raw data were analyzed using the SPSS 17.0 software package (SPSS Inc., Chicago). Homogeneity of variance was tested by using Levene’s test, and differences between means were determined using Duncan’s test (P < 0.05). One-way ANOVA was used to analyze differences in dodder biomass and irradiance between the native subcommunity and the parasitized *H. scandens* subcommunity as well as differences in *H. scandens* biomass between parasitized and nonparasitized *H. scandens* subcommunities in the field. Differences in percent parasitism, irradiance, water content, C content, N content and P content between the native and *H. scandens* subcommunities in the pot experiment were also determined by one-way ANOVA. Two-way ANOVA was used to determine the effects of origin (native vs. exotic) and density (low vs. high) on the percent parasitism by dodder in the different native host treatments. To assess the primary factors inducing the parasitic preferences of the dodder, a correlation matrix was constructed using Pearson’s linear correlation to assess the relationships among the variables (percent parasitism, light irradiance and plant water, C, N and P contents). Then stepwise multiple regression analysis was carried out with light irradiance and plant water as well as C, N and P contents as independent variables and percent parasitism as the dependent variable. Multicollinearity of the results was also checked by examining the variance inflation factors (VIF) of all predictor variables.

## Data Availability

The datasets generated during and/or analysed during the current study are available from the corresponding author on reasonable request.
